# Retinal migraine

**Published:** 2020

**Authors:** Bogdan Marius Istrate, Crisanda Vîlciu, Carmen Răgan

**Affiliations:** * University of Antwerp, Belgium; ** “Fundeni” Clinical Institute, Bucharest, Romania; “Mareș-Bulandra” Neurology Clinic, Bucharest, Romania; *** “Elias” Clinical Hospital, Neurology Clinic, Bucharest, Romania

**Keywords:** migraine, retinal blood vessels, eye vascular network, monocular visual loss

## Abstract

Retinal migraine is usually defined by transitory attacks of fully reversible monocular visual loss, mostly with aura. An accurate diagnostic can be completed based upon the International Classification of Headache Disorders-2 (ICHD-2) criteria. In view of this, we summarized some clinical features, treatment principles, complications, prognosis and prophylaxis.

## Introduction

Retinal migraine is an ophthalmo-pathological condition described as a transient monocular scotoma or vision loss, being accompanied or followed by a headache. The timing, the intensity and the aura (if present or preceded), may differ from person to person. Among other causes, apparently, the major one stays the ischemia or vascular spasm in, or behind the affected eye. A distinction should be noted, when a confusion between the terms “retinal migraine” and “visual migraine” arises. Visual migraine results from the cortical spreading depression and is denominated as scintillation scotoma. Retinal migraine is a rare retinal disorder. The symptoms are usually transient, but the pathophysiological mechanisms still remain not completely elucidated [**1**,**2**].

## Pathophysiological effectors and mechanisms

Substance P, nitrous oxide, calcitonin generated peptides have been suspected as chemical effectors in the possible pathophysiological mechanisms of retinal migraine, by exercising a non-desired effect leading to the plasma extravasation, neurogenic inflammation, vasodilatation. Other neuro-ophthalmological structures involved are: periaqueductal gray (PAG), locus coeruleus (LC), dorsal Raphe nucleus (DRC), retinal vasculature, and activation of the retinal-thalamic-visual pathway [**3**].

**Fig. 1:**
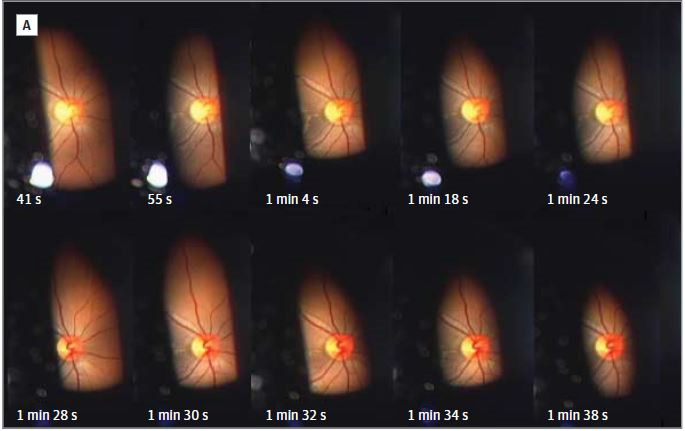
Video-assisted fundoscopy recorded for 1 min. 48 sec during the migraine attack. Dynamic changes in the retinal artery and veins can be detected. ***Source:** Ota I, Kuroshima K, Nagaoka T. Research Letter. Fundus video of retinal Migraine. JAMA Ophthalmology. November 2013; 131(11): 1481-1482 [**6**]*.

**Fig. 2:**
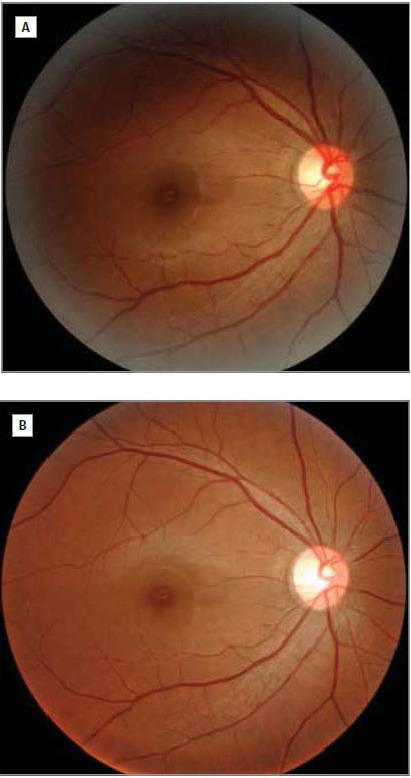
Fundoscopy of the eye, immediately after the attack onset (A), and at one-month distance after the attack (B). ***Source:** Ota I, Kuroshima K, Nagaoka T. Research Letter. Fundus video of retinal Migraine. JAMA Ophthalmology. November 2013; 131(11): 1481-1482 [**6**]*.

On the contrary, photophobia in migraine may start in cone-driven retinal pathways, exerting a hypersensitive-excitation effect on light-sensitive thalamic neurons. Photophobia is aggravated by the light-intensity dependence, when the eyes of the patients are exposed to different wavelengths of visible light. Fundoscopy (**Fig. 1**) and fluorescence angiogram expose a delayed tilling or occlusion of the central retinal artery and its branches, (**Fig. 2**) with, either normal ciliary circulation or irregular/ discontinuous choroidal defects and capillary leakage flux [**4**]. The vascular theory still remains doubtful due to the complexity of retinal vascular supply. Retina has a binary circulation: central retinal artery supplies, inner retinal layers. Those microstructures lack adrenergic innervation, maintain sensory-nerves and are auto-regulated [**5**].

## Symptomatology

When aura is present: flashing, sparkling, twinkling lights (scintillations). Non-aura: blind spot, a partial loss of vision, temporary blindness, scotoma. A retinal migraine attack begins with monocular visual symptoms, afterwards when relaxation time of the blood vessels is manifested, blood flow resumes and sight returns.

## Diagnostic tools and laboratory tests

They should be directed and based on the patient’s medical history and physical exam. Some laboratory blood markers, such as platelet count, coagulogram, homocysteine and protein S (optional), can be precious adjuvants as diagnostic tools. Tourniquet or capillary-fragility test (Rumpel-Leede test/ Hess test) can sometimes be a good option too. Optical coherence tomography, retinal oximetry, scanning laser ophthalmoscopic angiography, Doppler studies in order to investigate fundoscopy, visual field examination during the attack are also useful options.

## Reported complications

 - reversible and irreversible central retinal artery occlusion

- retinal infarction

- branch retinal artery occlusion

- retinal hemorrhages and disc edema

- ischemic optic neuropathy

- choroidal ischemia

- dilatation of retinal veins

- vitreous hemorrhage

- retinal pigmentary changes

- stroke

## Treatment

Analgesics and nonsteroidal anti-inflammatory drugs, caffeine, treatment with triptans, ergotamine compounds, may be a favorable option. Triptans and ergotamines, both exert an effect by stimulating the serotonergic receptors in the cerebral and cardiac vasculature. They should be instituted as therapy within 24h of each other [**7**].

## Prevention

It is well documented that visual disturbances caused by retinal migraine attack disappear without treatment within one hour or less. People performing activities that require clear vision, when a retinal migraine occurs, need to stop the activity and relax until the vision returns to normal, preferably in a dark or a semi obscure good freshly aerated room. If driving, they should park on the side of the road and wait for the vision disturbances to pass completely. They should also avoid common migraine triggers and stress, and they should get a regulated sleep and a healthy nutrition.

## Conclusions

Retinal migraine is a challenge and sometimes a pitfall before being diagnosed. Aura, is the most essential characteristic, but most cases labeled as “retinal migraine”, are not migraines. Sometimes, a vasculo-allergic migraine can be underdiagnosed or overdiagnosed as a retinal migraine. On the other hand, monocular visual phenomena typically originate in the retina, choroid and optical nerve. It is believed that retinal vasospasm initiates transient monocular visual loss, being the most plausible explanation. Optic nerve infarction and retinal infarction can occur due to the retinal vascular changes and the particularities during the migraine attack. Taking into account that vasospasm is the most common cause of the symptoms and the use of aspirin has its own risk and has been reported not very effective, the adequate use of verapamil and nifedipine, should constitute a good treatment option. No drug trial has been reported in retinal migraine, this being the reason why the treatment should be prescribed and orientated according to each patient’s needs. An inter-disciplinary consult of a neurologist and an ophthalmologist is a wise prerequisite.
